# “Dentists’ and dietitians’ recommendations of snacks and dental caries experience among kindergarteners”

**DOI:** 10.4317/jced.53092

**Published:** 2016-12-01

**Authors:** Masahiro Heima, Tanya Ranginwala, Sorin Teich

**Affiliations:** 1 Assistant Professor, Department of Pediatric Dentistry, School of Dental Medicine, Case Western Reserve University; 2 Assistant Professor, School of Dental Medicine, Case Western Reserve University; 3 Assistant Professor, Department of Comprehensive Care, School of Dental Medicine, Case Western Reserve University

## Abstract

**Background:**

Parents receive diet recommendations for their children from dentists and dietitians, but a conflict of diet suggestions has been reported. This research was conducted to investigate dental caries experiences in children consuming snacks that were recommended by dentists and/or dietitians.

**Material and Methods:**

A total of 442 kindergarteners under went dental examinations, and their caregivers filled out questionnaires. Snacks were sorted by name. Three dentists and three dietitians determined whether they would recommend these snacks. The snacks were divided into four categories: snacks recommended by both groups, snacks recommended by neither, snacks recommended only by dentists, and snacks recommend only by dieticians. Children were assigned to particular groups based on their primary snack consumption. The children’s caries experiences (dft) were compared among the four groups.

**Results:**

The agreement level on the recommended snacks between dietitians and dentists was moderate (Kappa=0.43). Thirty-nine snacks were identified; 13 recommended by neither, 4 recommended by dietitians, 7 were recommended only by dentists, and 15 were recommended by both. The mean (standard deviation) of dft amongthe children was 4.66 (3.81), 2.66 (3.17), 3.21 (3.37), and 4.02 (4.02), and respectively. The ANOVA and Tukey post-hoc tests indicated that children who consumed snacks recommended only by one professional, dietitian or dentist, have significantly fewer dental caries than children who consumed snacks recommended by neither professional. (ANOVA: F=4.494, *p*=0.004, Tukey post-hoc test: *p*=0.007 and *p*=0.046, respectively).

**Conclusions:**

Dentists can recommend snacks that are nutrient dense, even though it contains sucrose.

** Key words:**Child, dental caries, snacks, dentist, nutritionists.

## Introduction

The World Health Organization addresses the roles and responsibilities of both dietitians and oral health care professionals in promoting oral health and the consideration of diet in the prevention of oral diseases (1). In the United States, both the Academy of Nutrition and Dietetics, and the American Dental Association have also recognized the need for collaboration in service, education, and research to improve oral health (2,3). These organizations recommend that practitioners provide diet counseling and instructions for prevention of dental caries (3,4). Thus, caregivers of children receive dietary information from both dietitians and dentists. However, a conflict between these professionals’ diet counseling has been observed, (2,5) creating potential confusion in care givers of children.

Shah *et al.* (5) reported that dietitians are mainly concerned with the connection between nutrition and general health, while dentist’s consultations are mainly focused on the effects of food on the oral health, this leads to a conflict between dietary messages. However, effects of this conflict in diet consultation on dental caries have not been reported. This gap affects the education of students concerning diet, and interaction between practitioners and children’s caregivers about snack consumption with respect to nutrition and dentistry.

The purpose of this study was to investigate diet consultation on dental caries in children.

## Material and Methods

A dataset, which was utilized in this secondary data analysis, has been generated for a randomized clinical trial study. In the original study, 564 kindergarteners and their caregivers were recruited in five inner-city schools in one city in Northeast Ohio, in the United States, to investigate the effect of xylitol in preventing dental caries (6). The children were given dental examinations and their caregiver was asked to complete a questionnaire, which consisted of questions about their child’s snack characteristics and consumption information.

-Development of the Snacks List

 In the questionnaire, the caregivers were asked an open-ended question about children’s snack consumption, “What are the three most common snack foods that your child eats during the day?” For the purpose of this study, three blinded dentists and three blinded dietitians, independently evaluated if each snack was “recommended” or “not recommended” for children. Each snack was determined to be either “recommended” or “not recommended” by majority decision within dentist or dietitian groups. The inter-rater agreements within a profession and inter-rater agreements between both groups of professionals were calculated.

-Dental examination: In the original study, the International Caries Detection and Assessment System (ICDAS) was used by 3-4 pediatric dentists and residents who were trained and calibrated. The inter- and intra-rater reliability of examiners was 0.77-0.98 and 0.93-1.00, respectively (7). Portable dental chairs (UltraLite Patient Chair, DNTLworks Equipment Corporation, Inc., Centennial, Colorado, USA) equipped with compressed air and halogen lights were used for the dental exams. According to the IC-DAS protocol, radiographic examination was not used (8). Children’s surface level dental caries experiences were recorded and the number of primary teeth with either decayed or filled teeth (dft) was calculated.

-The effect of the conflict of snack recommendations on the children’s dental caries experiences.

The preliminary exploratory data analyses test for the relationship among children’s dental caries experiences and the first snack to the third snack consumption. Although significant differences of dft among the first to third snacks were not observed, the largest differences of the mean dft were between the first snacks, which were recommended by both professionals and the first snacks, which were not recommended by either professional (4.02 and 4.66, respectively). To investigate the effect of the conflict of snack recommendations on the children’s dental caries experiences (dft), we used the first snacks, which were reported by their caregivers.

All children were separated into four groups based on their first snacks:“Not Recommended by either professional”, “Recommended only by dietitian”, “Recommended only by dentist”, and “Recommended by both professionals”. ANOVA was utilized to compare dft among multiple groups. Tukey post-hoc test was utilized for Multiple comparison of dft for each pair of groups.

## Results

From all recruited subjects (564), 423 (75%) pairs of kindergarteners and their caregivers completed both the dental examination and the questionnaire of the common snacks. These data were then analyzed.

-Characteristics of subjects: The gender ratio (boy/girl) of the kindergarteners was 46/54 and 85% of their caregivers were mothers. The caregivers’ high school completion rate was 76.9%. Of the children 96% were eligible for the federal free/reduced cost school lunch program, which is available if the household’s gross income is not greater than 130% of the Federal Poverty Guidelines. Most children (94%) were African-American.

There were no significant differences in descriptive characteristic analyses for children’s gender ratio, caregivers’ education level, and caregivers’ marriage status, among the four groups.

Snack list: Thirty-nine snacks were identified based on the snack question. Cohen’s Kappa inter-rater agreements test within dietitians and dentists indicated a moderate level of agreement: 0.36 to 0.45 and 0.43 to 0.78, respectively (9).  Table 1 shows the examples of snacks and recommendations by professionals. Twenty-eight (71.8%) kinds of snacks were agreed upon dentists and dietitians (15 snacks recommended and 13 snacks not recommended) while 11 (28.2%) kinds of snacks were disagreed upon (recommendation conflict). Among these, four kinds of snacks were recommended only by dietitians, while seven kinds of snacks were recommended only by dentists.

**Table 1:**
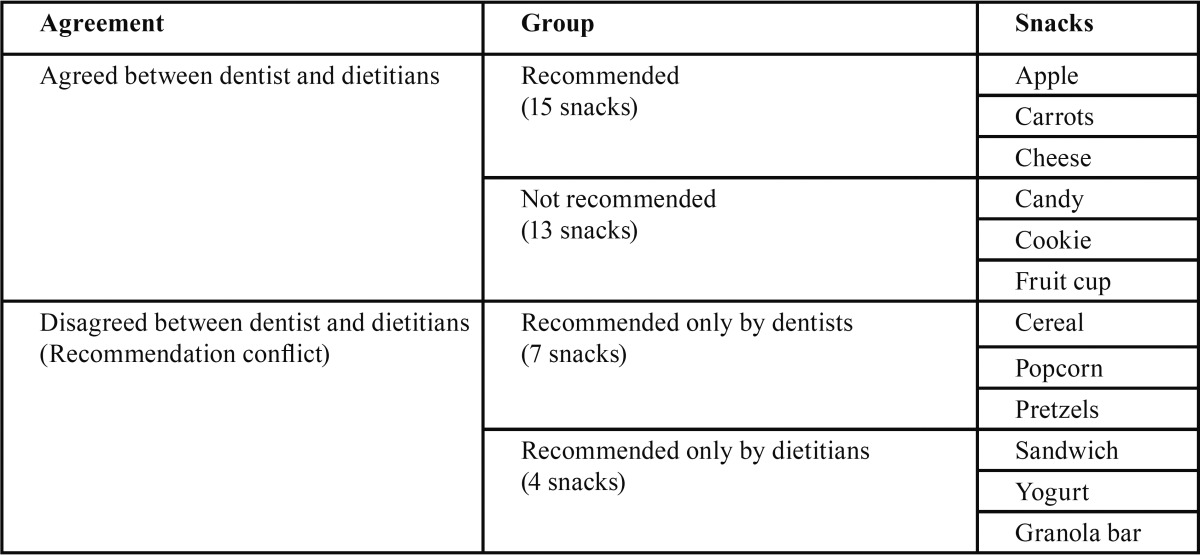
Example of snacks and professionals’ recommendations.

-Comparisons of children’s dental caries experiences between snack recommendations:  Table 2 shows that there was no signifi-cant relationship between dental caries experience (dft) and snack recommendations between professionals.

**Table 2:**
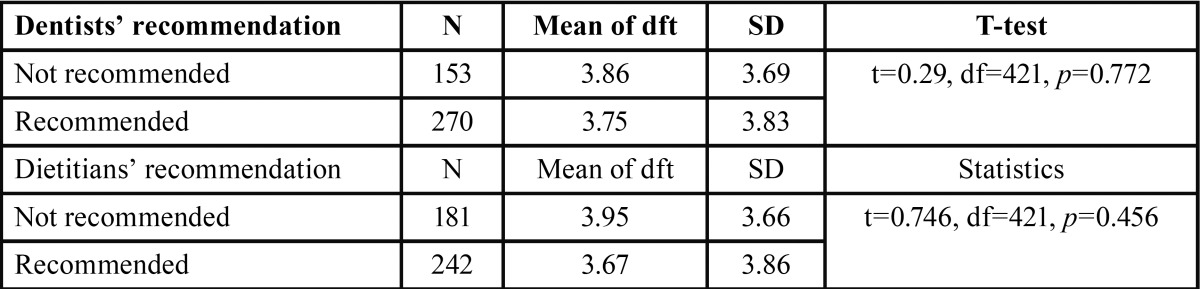
Dental caries experience by recommendations and each professional.

 Table 3 shows means of the children’s dental caries experiences with standard deviations; ANOVA indicated there were significant differences among the groups (ANOVA: F=4.494, *p*=0.004), and  Table 4 shows Tukey post hoc multiple comparisons. The results indicate that children who consumed snacks recommended by either dietitians or dentists had significantly fewer dental caries than did children who consumed snacks not recommended by either professional (*p*=0.007 and 0.046, respectively.)

**Table 3:**
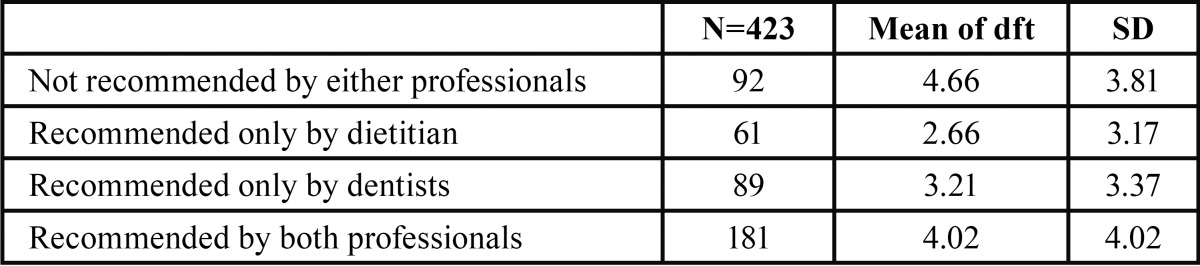
Children’s dft by recommendations.

**Table 4:**
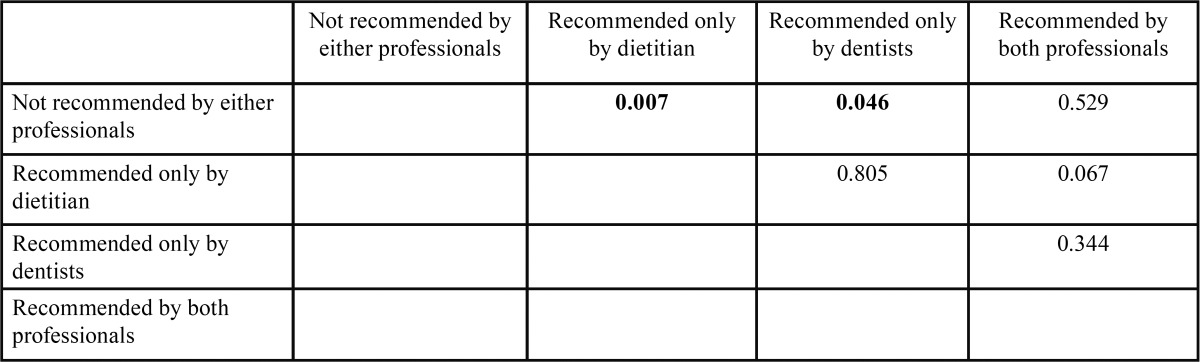
Multiple comparison of dft by Tukey post-hoc test.

## Discussion

This is the first investigation of the effect of conflict of diet recommendation of children’s snack between dietitians and dentists on children’s dental caries experiences. Surprisingly, we found that children who consume snacks recommended by only dietitians have the least number of dental caries; children who consume snacks recommended by only dentists had slightly more dental caries. Furthermore, there is no significant difference between children consuming snacks recommended by both professional groups or not recommended by either group.

-The conflict in the recommendations of snacks: We anticipated a conflict of snack recommendations between dietitians and dentist according to the previous study (5). The dietitians made recommendations based on the “balance of nutrition” (amount and variety of nutrients including protein, carbohydrates, fat, etc.) and the dentists made recommendations based on cariogenicity (5). We observed in the interviews with the dietitians and dentists, that there are multiple factors involved in making recommendations including, each professional’s perception, training differences, and clinical experience, all of which lead to conflicts in diet consultations. In fact, Threlfall asked dentists how they inform patients for prevention of dental caries and reported that there was a large variance in the dietary counseling with in the dental community, although dentists know the basic principles of diet counseling (10). For example, one of the dentists mentioned that her impression of sandwiches was a chocolate peanut butter sandwich; while one of the dietitians said that her impression of it was a ham and lettuce sandwich.

-The effect of the recommendation on children’s dental caries experiences: Children who consumed snacks recommended by dietitians had the fewest dental caries. The snacks recommended by both professionals were non-sucrose containing such as fresh fruits and vegetable. Snacks not recommended by both professionals were high dose, sucrose-containing snacks such as candy and diced fruits with syrup. Snacks recommended by either one of the professional groups had moderate sucrose level, such as bread, cereal and yogurt. Even though professionals’ opinions were mixed, children who were reported consuming these types of snacks by their caregiver had fewer dental caries.

-Limitations: The snack information was extracted from self-reported questionnaires, which were administered by a dental research team; therefore, asocial desirability biasmay have been included in responses. Caregivers need to pay attention to actual diets rather than responding in a wish-fulfillment pose.

The snack list provided only the name of snacks provided by the caregivers; it did not provide any ingredients of the snacks. The professionals had to decide whether the snack was “recommended” or “not recommended” based on their assumptions of the composition of the snacks.

We asked local dentists and dietitians to make their recommendations; only three professionals were in each group. Their recommendations might not represent the professional consensus. However, this study was the first to compare children’s dental caries experiences by snacks, including recommendations by two different professional clinicians.

## Conclusions

This being the case, dentists can recommend snacks that are nutrient dense, even though it contains sucrose.
